# Supplementary material for: A role for tectorial membrane mechanics in activating the cochlear amplifier
Source: Sci Rep. 2020 Oct 19;10:17620. doi: 10.1038/s41598-020-73873-9 (PMC7573614; doi:10.1038/s41598-020-73873-9)
Supplement: Supplementary file 1 — Supplementary Information. [file 41598_2020_73873_MOESM1_ESM.pdf]

Supplemental Information: A role for tectorial membrane mechanics in activating the cochlear amplifier

Amir Nankali[1], Yi Wang[4], Clark Elliott Strimbu[4], Elizabeth S. Olson[3,4], and Karl Grosh[1,2*]

[1]Department of Mechanical Engineering, University of Michigan, Ann Arbor, MI, 48109, USA
[2]Department of Biomedical Engineering, University of Michigan, Ann Arbor, MI, 48109, USA
[3]Otolaryngology, Head and Neck Surgery, Columbia University, New York, New York
[4]Biomedical Engineering, Columbia University, New York, New York
[*]grosh@umich.edu

Exploration of possibility that phase cancellation contributed to sub-CF notch and phase shift

We wanted to consider the possibility that the sub-CF notch might be due to phase cancellation of currents from local and distant cochlear locations, rather than a local electromechanical effect. We have explored this possibility in two ways. Our first approach was to exercise our full numerical model, where we can turn off longitudinal coupling of the electrical current (by artificially increasing the resistance in the scalae). In our model, the electrical response is computed using multiple cables (ST, SV, SM) coupled to the active cochlear model (see Fig.S1A and Ref.[1] for the detail of the cable model). In the case when there is no current spread, propagation of current beyond each cross section is not possible and phase cancellation is likewise not possible. With a current spread of zero the notch in the magnitude and the phase change persists (see Fig. S1B-C). When current spread is turned back on (using the normal model parameters) the notch is slightly diminished in depth compared to the "no cables" model. A notch appears above the CF where the phase is varying rapidly, showing that phase cancellation can occur in the model. Therefore, theory predicts current spread is not the main factor for the sub-CF notch and associated phase change.

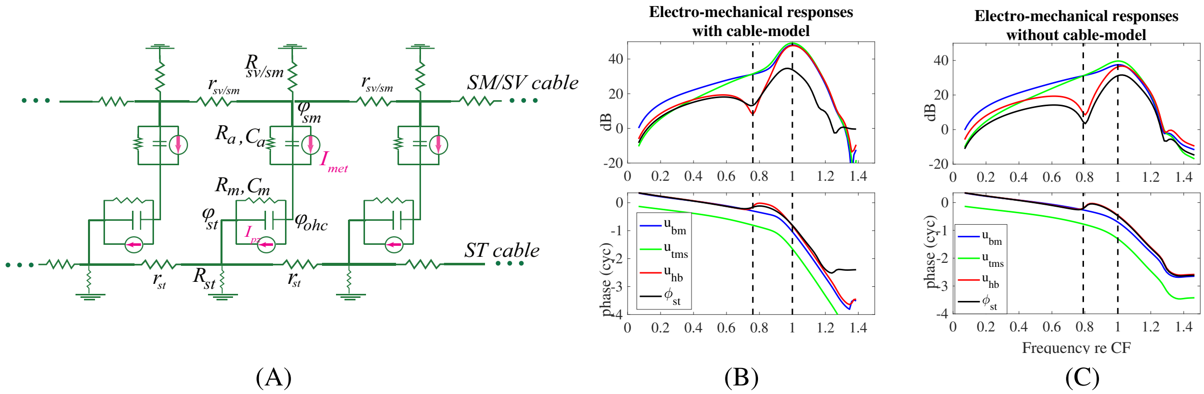

Figure S1. Using the full cochlear model of this paper to examine the possibility that the sub-CF phase shift of voltage relative to BM displacement, and often-accompanying amplitude notch, are due to phase cancellation from distant sources. (A) Electrical network of the OHCs (see Fig 3C) coupled along the cochlea using different cable models on the SM/SV and ST. (B) The full model responses with the cable model. (C) The model responses without the cable model (i.e, no current spread along the cochlea was allowed). The phase shift and voltage notch are present in both cases, indicating they are not due to phase cancellation from interacting electric fields.

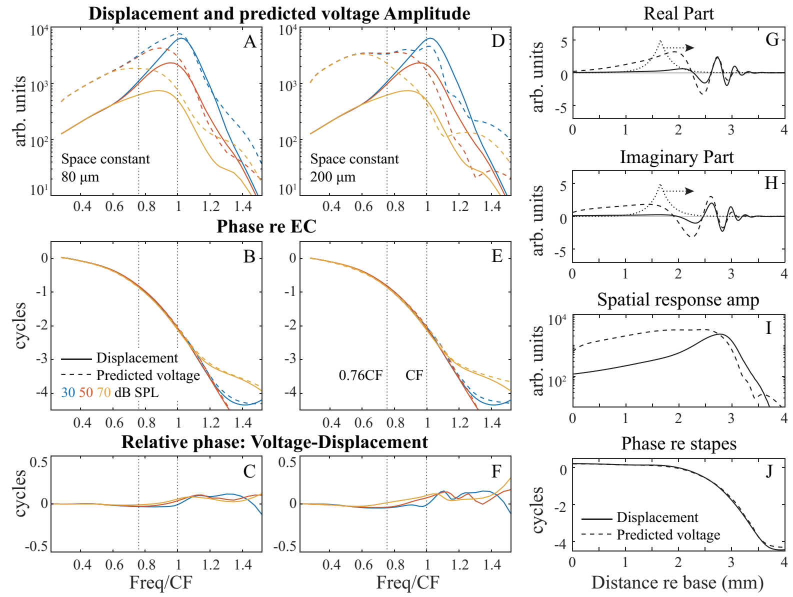

Figure S2. Using the experimental data to explore the potential effect of current spread on the sub-CF notch and phase shift. Two different exponential decay lengths are used, 80 μm and 200 μm. 80 μm was used in Dong & Olson[2] to fit their data, and 200 μm is included for illustration because it allows for a larger contribution from distant sources. In panels A-F the color code represents the SPL applied (see panel B) while the displacement field is given by solid lines and the predicted voltage is shown using dashed lines in A,B,D,E. The horizontal axis in A-F is frequency/CF. In G-H the point spread function is shown using a dotted curve and the horizontal arrow represents the convolution operator with the displacement field (solid line). The result of the convolution operation is the predicted voltage field shown using a dashed line. I-J The amplitude and phase are shown for one frequency, versus longitudinal location. The location data are cast back into the frequency domain for the voltage predictions in A-F. Phase cancellation produces supra-CF amplitude notches, but not sub-CF notches. Their location varies with frequency and amplitude. A sub-CF phase shift is not produced.

Second, as in[2], we used a cable model (that paper's Fig. 7 and 8) to explore the effect of current spread. This approach was also used in Ref.[3] and several other works[4,5]. In the cable model OHC transducer current was assumed to be linearly proportional to BM displacement (a simplifying assumption). Using a post-processing technique, the frequency response of the BM displacement (transducer current) at a single location is transformed to a spatial dependence according to a scale invariant approximation (e.g., following Olson, et al.[6]). The transducer current is then spatially convolved with the current point spread function (Green's function) with a space constant (80 and 200 microns were explored) to obtain the spatial dependence of the voltage field. We assume a space constant larger than the 42 μm value indicated by[3] for the space constant within the OCC; the larger value is a conservative approximation as it would induce greater phase cancellation. The process and result is in Fig. S2. No sub-CF notches were introduced in the voltage. Based on the results of Figs. S1 and S2, phase cancellation due to current spread does not produce the sub-CF phase shift and amplitude notch in voltage.

References

1. Ramamoorthy, S., Deo, N. V. & Grosh, K. A mechano-electro-acoustical model for the cochlea: response to acoustic stimuli. *The J. Acoust. Soc. Am.* **121**, 2758–2773 (2007).

2. Dong, W. & Olson, E. S. Detection of cochlear amplification and its activation. *Biophys. J.* **105**, 1067–1078 (2013).

3. Fridberger, A. *et al.* Organ of corti potentials and the motion of the basilar membrane. *J. Neurosci.* **24**, 10057–10063 (2004).

4. Patuzzi, R. A model of the generation of the cochlear microphonic with nonlinear hair cell transduction and nonlinear basilar membrane mechanics. *Hear. Res.* **30**, 73–82 (1987).

5. Cheatham, M. A., Naik, K. & Dallos, P. Using the cochlear microphonic as a tool to evaluate cochlear function in mouse models of hearing. *J. Assoc. for Res. Otolaryngol.* **12**, 113–125 (2011).

6. Olson, E. S., Duifhuis, H. & Steele, C. R. Von békésy and cochlear mechanics. *Hear. Res.* **293**, 31–43 (2012).
